# Assessing outcomes of genetic selection panels to predict marbling in crossbred beef cattle

**DOI:** 10.1093/tas/txaa077

**Published:** 2020-06-05

**Authors:** Tanya M Weber, Brianna J Buseman, James A Nasados, Jessica M Lancaster, Jessie B Van Buren, Jaxon H Smart, Phillip D Bass, Gordon K Murdoch, Kizkitza Insausti, Michael J Colle

**Affiliations:** 1 Department of Animal and Veterinary Science, University of Idaho, Moscow, ID; 2 Agricultural Engineering School-IS FOOD, Public University of Navarra, Pamplona, Spain

**Keywords:** beef quality, genetic panels, Igenity, marbling, PredicGEN

## Abstract

The objective of this study was to evaluate the effectiveness of genetic panel marbling indexes [Igenity (IT) and PredicGEN (PG)] to predict marbling and tenderness of crossbred cattle. Steers (*n* = 23) were harvested at the University of Idaho Meat Science Laboratory, and blood samples were submitted to Neogen and Zoetis for genetic panel analysis. Forty-eight hours postharvest, one boneless strip loin was collected from each carcass, and six 2.54-cm thick steaks were cut from each strip loin. Steaks were aged for 14 and 21 d and assigned to consumer sensory evaluation or Warner–Bratzler Shear Force (WBSF) analysis. Results were analyzed using the Mixed Model procedure of the Statistical Analysis System (SAS Institute, Inc., Cary, NC). Carcasses were grouped by marbling index score into Low IT (IT indexes 3–6; *n* = 16; marbling score (MS) = 410), High IT (IT indexes 7–10; *n* = 7; MS = 496), Low PG (PG index <50; *n* = 9; MS = 398), or High PG (PG index ≥50; *n* = 14; MS = 458). Mean MS was observed to be greater in High IT steaks than Low IT (*P* < 0.01) and greater in High PG steaks than Low PG (*P* = 0.01). There was a trend observed in WBSF between IT marbling groups (*P* = 0.06); however, no difference in WBSF was observed between PG marbling groups (*P* = 0.83). Consumers did not report differences between IT marbling groups in terms of acceptability (*P* = 0.99) or tenderness (*P* = 0.24). Additionally, consumers could not detect differences between PG marbling groups in terms of acceptability (*P* = 0.75) or tenderness (*P* = 0.40). Consumers consistently preferred Choice steaks over Select steaks in terms of acceptability (*P* = 0.02) and tenderness (*P* = 0.02). In conclusion, though consumers were not able to tell the difference between steaks from each of the genetic panels, using genetic panels to predict marbling, in conjunction with proper nutrition and handling practices, could be a beneficial tool to producers making decisions about retaining ownership at the feedlot.

## INTRODUCTION

Marbling is defined as intramuscular fat (Ferguson, 2004) and is influenced by nutrition (Pethick et al., 2004), management (Meyer et al., 2005; Park et al., 2018), and genetics (Utreta and Van Vleck, 2004; Albrecht et al., 2011). Marbling deposition has been linked primarily to the leptin gene (Buchanan et al., 2002; Geary et al., 2003; Yamada et al., 2003; Bonnet et al., 2007). DeVuyst et al. (2007) observed that cattle with the homozygous “fat” leptin genotype were more valuable than other genotypes. It has been well documented that improvements in marbling improves tenderness, both objectively (McBee and Wiles, 1967; Luchak et al., 1998) and via consumer perception (Millar, 1994; Li et al., 2006). It is for these reasons that beef packing facilities utilize services of United States Department of Agriculture (USDA) Agriculture Marketing Service (AMS) grading personnel to assign carcasses a USDA quality grade. This allows beef cattle producers to receive a premium, or avoid a discount, for carcasses with high degrees of marbling while allowing packers to apply discounts for carcasses with poor marbling (USDA Livestock, Poultry, and Grain Market News Division, 2020).

Additionally, increased marbling improves palatability traits of beef (Smith et al., 1987; Magolski et al., 2013; Corbin et al., 2015; Lucherk et al., 2016). Thompson et al. (2014) determined that phenotypic traits were indeed correlated with their genetic panel values, but these tests would be a more economically important test to use for replacement breeding stock, not necessarily when separating animals at the feedlot. To date, no research has been published comparing Warner–Bratzler shear force (WBSF) and consumer sensory panel data with genetic information derived from commercially available genetic tests on crossbred cattle.

The objective of the current study was to evaluate the effectiveness of genetic panel marbling indexes [Igenity (IT) and PredicGEN (PG)] to predict marbling and tenderness of crossbred beef steers. The hypothesis was that crossbred steers with higher IT and/or PG marbling indexes would produce carcasses with more marbling and that are more tender than crossbred steers with lower IT or PG marbling indexes.

## MATERIALS AND METHODS

### Human Subject Participation in Consumer Sensory Panel

The University of Idaho Institutional Review Board certified this project as exempt.

### Obtaining DNA samples

Crossbred steers (Angus × Hereford × Simmental; *n* = 23) were harvested under inspection at the University of Idaho’s USDA-FSIS inspected Vandal Brand Meats Laboratory. Blood (1 mL; IACUC 2017–32) was pipetted onto blood cards, one from Zoetis (PredicGEN) and one from Neogen (Igenity), to be analyzed for DNA analysis.

### Carcass Characteristics

Marbling score (MS) on each carcass was determined visually by trained University of Idaho research team members using USDA quality grading standards at 24 h postmortem and 1 h after the carcass was ribbed between the 12th and 13th ribs. Quality grade was assigned to carcasses using MS (high Select: MS 350–399; low Choice: MS 400–499; USDA/AMS/LPSP, 2017). Also at 24 h postmortem, yield grade (YG) was calculated by trained University of Idaho research team members to carcasses using the formula 2.50 + [2.50 × adjusted backfat thickness (BF), inches] + (0.20 × percentage of kidney, pelvic, and heart fat) + [0.0038 × hot carcass weight (HCW), pounds] − [0.32 × ribeye area (REA), square inches] (USDA 2017).

### Steaks

Boneless strip loins (IMPS #180; *n* = 23) were fabricated from each carcass at 48 h postharvest and vacuum packaged for subsequent analysis. Carcasses were grouped by marbling index score into Low IT (IT indexes 3–6), High IT (IT indexes 7–10), Low PG (PG index <50), or High PG (PG index ≥50). Steaks were further grouped by their quality grade. Six 2.54-cm thick steaks were cut from the anterior end of each strip loin and randomly assigned to one of six groups for evaluation. Steaks were assigned to either a 14- or 21-d postmortem aging period followed by a consumer sensory panel (SP; 14-d IT SP, 21-d IT SP, 14-d PG SP, and 21-d PG SP) or WBSF (14-d WBSF and 21-d WBSF) analysis. Steaks were vacuum packaged individually and aged (0 ºC) for their respective amounts of time before being frozen at −20 ºC until subsequent analysis could occur.

### Cooking

Steaks were thawed for 24 h at 0 ºC. They were then cooked on a clam-shell style Cuisinart grill (Cuisinart Griddler Deluxe Model GR-150) that was set to 204 ºC on both grill plates to a target peak internal steak temperature of 71 ºC. Temperatures were monitored using a type K thermocouple (93230-K EconoTemp, Cooper-Atkins, Middlefield, CT) placed at the approximate geometric center of each steak. The steaks were removed from the grill at 65 ºC; temperature was monitored until it began to decline, and the peak temperature was recorded.

### Warner–Bratzler Shear Force

Steaks were cooked as described above. The cooked steaks were allowed to cool to room temperature on a tray. Once cooled, steaks were weighed again to determine cook loss. At least six cores were obtained from each steak parallel to the muscle fibers orientation, taking care to avoid connective tissue and excess fat. Steaks were cored using a Shop Fox W1667 8-1/2” oscillating drill press with a 1.27-cm diameter coring bit attachment. All cores were sheared using a Warner–Bratzler Meat Shear (G•R Manufacturing, CO, Manhattan, KS, BFG 1000N) machine and the peak shear force of each core was recorded. The average of the shear force values for all cores from each respective steak was calculated and was analyzed to determine the WBSF of each steak.

### Consumer Sensory Panel

Steaks were assigned in an incomplete block design to a cooking order and cooked as described above. Panelists were given a demographics cover page (Table 1) and a questionnaire that asked them to rank each sample on an unstructured scale of 1–10, with 1 being the least favorable in its category and 10 being the most favorable in its category. The rankings were assigned based on each panelist’s opinion of the steak’s tenderness, flavor, juiciness, and overall acceptability. Each panelist was given five samples at the same time and asked to try them in their randomly assigned sampling order. Samples were cut into 1.27- × 1.27- × 2.54-cm cubes. Panelists (*n* = 92) were given water and salt-free soda crackers to cleanse their palette between samples.

**Table 1.** Demographics of consumer panelists (*n* = 92)

	*n*	%
Age		
18–29	66	72
30–39	11	12
40–49	2	2
50+	13	14
Gender		
Male	49	53
Female	43	47
Beef meals/wk		
0–1	10	11
2–4	52	57
5–7	22	24
8+	8	9
Most consumed		
Ground	62	67
Roast	6	7
Steak	23	25
Other	1	1

### Statistical Analysis

Data were analyzed using the Mixed Model procedure assuming a normal distribution. Within each model, dependent variables were WBSF, MS, consumer perception of overall acceptability, tenderness, juiciness, and flavor. Additionally, aging treatment, genetic panel marbling group, and quality grade were fixed effects. The relationship between YG, WBSF, MS, and genetic panel index scores for marbling was assessed using Pearson correlation analysis. Significance was determined at *P* < 0.05, and trends were determined at *P* < 0.10. For significant fixed effects, means were separated using pair-wise comparisons. All statistical analyses were carried out using SAS V9.4.

## RESULTS

### Carcass Characteristics

Carcass characteristics are summarized in Table 2. Mean MS was greater in the High IT group than the Low IT group (*P* < 0.01). Additionally, mean MS was greater in the High PG group than in the Low PG group (*P* = 0.01). Mean MS was greater in Choice carcasses than in Select carcasses (*P* < 0.01). No difference was observed in mean HCW based on IT group (*P* = 0.49) or PG group (*P* = 0.28). Choice carcasses, however, were heavier than Select carcasses (*P* < 0.01). Carcasses that fell into the High IT group had greater REA than the Low IT group (*P* < 0.01). No difference was observed in mean REA between PG groups (*P* = 0.68) or quality grades (*P* = 0.43). Additionally, no difference was observed between mean BF between IT groups (*P* = 0.57), PG groups (*P* = 0.06), or quality grades (*P* = 0.18). Carcasses that fell into the High IT group had lesser calculated YG than Low IT carcasses (*P* = 0.047), and carcasses that fell into the High PG group tended to have lesser YG than Low PG carcasses (*P* = 0.09). No difference was observed in calculated YG between quality grades (*P* = 0.28).

**Table 2.** Carcass summary statistics*

Group	*n*	MS†	BF	HCW	REA	YG
By IT‡						
High	7	496 ± 18^a^	1.11 ± 0.12	383 ± 6	36.0 ± 1.0^a^	2.6 ± 0.2^b^
Low	16	410 ± 12^b^	1.19 ± 0.08	378 ± 4	32.8 ± 0.7^b^	3.1 ± 0.1^a^
By PG**						
High	14	458 ± 13^a^	1.05 ± 0.09	375 ± 5	33.9 ± 0.9	2.8 ± 0.2
Low	9	398 ± 17^b^	1.30 ± 0.09	383 ± 5	33.4 ± 0.9	3.2 ± 0.2
By quality grade						
Select	6	352 ± 16^b^	1.05 ± 0.13	369 ± 7^b^	34.0 ± 1.1	2.7 ± 0.2
Choice	17	464 ± 10^a^	1.24 ± 0.07	392 ± 4^a^	34.9 ± 0.6	3.0 ± 0.1
Overall	23	436	1.21	385	34.0	3.0

*Values represented as means ± SEM.

†MS: 350–399 = Select^+^; 400–499 = Choice^−^.

‡Igenity marbling group.

**PredicGEN marbling group.

^a,b^Within a group and column, means without a common superscript differ (*P* < 0.05).

Mean MS was positively correlated (*r* = 0.39) with PG marbling index scores (*P* < 0.01). Additionally, MS was positively correlated (*r* = 0.47) with IT marbling indexes (*P* < 0.01). Calculated YG was negatively correlated (*r* = −0.39) with PG marbling index scores (*P* < 0.01), while PG marbling index scores and IT marbling index scores were positively correlated (*r* = 0.55) with each other (*P* < 0.01).

### Warner–Bratzler Shear Force

Mean final off temperature of the steaks was 70.65 ± 0.30 ºC. There was a trend observed for the High IT group to have higher WBSF values than the Low IT group (*P* = 0.06; Table 3). No difference in WBSF was observed between PG marbling groups (*P* = 0.83; Table 4) or quality grades (*P* = 0.88; Table 5). No postmortem aging treatment effect (14 vs. 21 d) was observed (*P* = 0.16).

**Table 3.** Effects of Igenity marbling index score on palatability traits

Igenity marbling group				
Trait	Low (*n* = 16)	High (*n* = 7)	SEM	*P-*value
WBSF	2.76	3.21	0.21	0.06
Sensory traits (*n* = 92 panelists)				
Acceptability	6.6	6.6	0.2	0.99
Tenderness	6.3	6.6	0.2	0.24
Juiciness	6.0	6.3	0.2	0.20
Flavor	6.3	6.0	0.2	0.21

Scale, 10 = extremely tender, extremely juicy, extremely flavorful, and extremely acceptable, respectively; 1= not at all tender, extremely dry, dislike flavor extremely, and extremely unacceptable, respectively.

^a,b^Within a row, means without a common superscript differ (*P* < 0.05).

**Table 4.** Effects of PredicGEN marbling index score on palatability traits

	PredicGEN marbling group			
Trait	Low (*n* = 9)	High (*n* = 14)	SEM	*P*-value
WBSF	2.91	2.86	0.18	0.83
Sensory traits (*n* = 92 panelists)				
Acceptability	6.6	6.7	0.2	0.75
Tenderness	6.3	6.5	0.2	0.40
Juiciness	5.9	6.3	0.2	0.05
Flavor	6.2	6.2	0.2	0.99

Scale, 10 = extremely tender, extremely juicy, extremely flavorful, and extremely acceptable, respectively; 1 = not at all tender, extremely dry, dislike flavor extremely, and extremely unacceptable, respectively.

^a,b^Within a row, means without a common superscript differ (*P* < 0.05).

**Table 5.** Effects of quality grade on palatability traits

	Quality grade			
Trait	Select (*n* = 6)	Choice (*n* = 17)	SEM	*P-*value
WBSF	2.96	3.00	0.21	0.88
Sensory traits (*n* = 92 panelists)				
Acceptability	6.4^b^	6.9^a^	0.2	0.02
Tenderness	6.2^b^	6.7^a^	0.2	0.02
Juiciness	5.9^b^	6.5^a^	0.2	<0.01
Flavor	6.0	6.3	0.2	0.25

Scale, 10 = extremely tender, extremely juicy, extremely flavorful, and extremely acceptable, respectively; 1 = not at all tender, extremely dry, dislike flavor extremely, and extremely unacceptable, respectively.

^a,b^Within a row, means without a common superscript differ (*P* < 0.05).

### Consumer Sensory Panel

Mean final off temperature for consumer sensory analysis was 71.15 ± 0.22 ºC. Consumers were not able to detect differences between Low IT and High IT groups in terms of acceptability (*P* = 0.99), tenderness (*P* = 0.24), juiciness (*P* = 0.20), or flavor (*P* = 0.21; Table 3). They were also unable to detect differences between PG marbling groups in terms of acceptability (*P* = 0.75), tenderness (*P* = 0.40), or flavor (*P* = 0.99) (Table 4). However, there was a trend observed for consumers to consider steaks from the High PG group to be juicier than steaks from the Low PG group (*P* = 0.05). Consumers preferred Choice steaks over Select steaks in terms of acceptability (*P* = 0.02), tenderness (*P* = 0.02), and juiciness (*P* < 0.01; Table 5). Additionally, consumers were not able to detect any flavor differences between quality grades (*P* = 0.25). No aging treatment effect was observed for acceptability (*P* = 0.15), juiciness (*P* = 0.19), or flavor (*P* = 0.71). A trend was observed for consumers to prefer steaks aged 14 d (mean tenderness score = 6.6 ± 0.2) over steaks aged for 21 d (mean tenderness score = 6.2 ± 0.2) in terms of tenderness (*P* = 0.06).

## DISCUSSION

Steaks from the High IT group had significantly greater MS than steaks from the Low IT group. This is consistent with research conducted by Shackelford et al. (1994), Minick et al. (2004), and Utrera and Van Vleck (2004) showing high heritability of marbling. Additionally, High PG steaks had greater marbling than Low PG steaks. Zuidema et al. (2017) found a moderate correlation between the IT MS and the PG MS, which suggests that the two panels can share some similar single nucleotide polymorphisms (SNP) with a similar effect to evaluate marbling genotype. Furthermore, PG MS and IT MS were positively correlated with each other in this study, further leaning toward that conclusion. Some carcasses that fell into the High IT group, however, did not fall into the High PG group, and vice versa, which suggests that the SNP that are used between the panels are similar but not exactly equivalent. Furthermore, this suggests that panels differ, possibly in number of SNP and effects of markers evaluated. Additionally, marbling is a trait that is influenced by many different environmental factors, including nutrition (Pethick et al., 2004), management (Meyer et al., 2005; Park et al., 2018), climate (Tume, 2004), and time on feed (Spehar et al., 2009). This supports the observation of the present study, where there were Choice carcasses in the Low IT and Low PG groups and Select carcasses in both High IT and High PG groups. The SNP that are used in the tests are proprietary, so researchers can only speculate about which SNP are used. The objective of this study was not to evaluate the two panels, rather, it was to evaluate the effectiveness of the commercially available genetic panels in a way that a beef cattle producer might apply them profitably in their operation management.

Calculated YG was observed to be lower in High PG carcasses than Low PG carcasses, and a tendency was observed for YG to be lower in High IT carcasses than in Low IT Carcasses. This observation is unexpected as it has been observed that genetic improvement in YG has deleterious effects on quality grade (Thompson et al., 2015). Additionally, YG was negatively correlated with PG marbling index. These observations conflicts with earlier research by DeVuyst et al. (2011), who observed positive correlations between Igenity marbling index score and YG. Since both tests index scores were positively correlated with each other, it could be anticipated that the PG test would place animals into similar marbling groups as the IT test and, therefore, exhibit similar relationships with YG. Additionally, this observation conflicts with expectations that a greater YG would be positively correlated with marbling due to the greater fatness, which has been associated with cattle that have greater MS (Jones et al., 1990). This suggests that selection for greater genetic potential for marbling using either genetic panel has the potential to improve overall YG, further extending producers’ ability to conjure premiums from their animals through genetic management.

No differences were observed for WBSF between Low IT and High IT or between Low PG and High PG carcasses. There was, however, a trend for High IT steaks to have higher WBSF values, meaning tougher steaks, than Low IT steaks. This is not what was expected, given the observed relationship between marbling and tenderness (McBee and Wiles, 1967; Millar, 1994; Li et al., 2006), but the mean WBSF value of High IT steaks (3.21 kg) was still below the USDA *Certified Very Tender* threshold of <3.9 kg (ASTM, 2011). Additionally, no difference was observed for WBSF between Choice and Select carcasses. McBee and Wiles (1967), Millar (1994), Luchak et al. (1998), and Li et al. (2006), however, found a significant decrease in WBSF value as marbling units increased. Tenderness is influenced by multiple environmental factors, including cooler temperature (Locker and Haygard, 1963) and degree of doneness (Parrish et al., 1973). In the present study, all group means fell below the threshold for being considered Certified Very Tender (WBSF <3.9 kg; ASTM, 2011). Additionally, consumers were not able to detect differences between High IT and Low IT steaks in terms of tenderness, which aligns with Miller et al. (1995), who found that consumers were not able to detect differences of less than 0.5 kg of WBSF; the difference between the two IT marbling groups was 0.45 kg of WBSF.

Consumers were not able to detect differences between High IT and Low IT groups in terms of overall acceptability, juiciness, or flavor. This is likely because the difference between the two groups in terms of MS, though significant, still fell within the same USDA quality grade. The reason for this observation is likely because all the cattle, while crossbred, were genetically similar as they were all backgrounded in the same location. All the cattle used in the present study were finished in the same location, and they were implanted with trenbolone acetate (Synovex One, Zoetis, Kalamazoo, MI) at the beginning of the finishing period. Additionally, consumers were not able to tell the difference between High PG and Low PG in terms of acceptability, tenderness, or flavor, but they tended to prefer High PG steaks over Low PG steaks based on juiciness. This is likely because the mean MS difference between the two groups translated to high Select and low Choice USDA quality grades, and consumers are known to prefer the juiciness of Choice steaks over Select steaks (Corbin et al., 2015).

Consumers preferred Choice steaks over Select steaks in terms of acceptability, tenderness, and juiciness. This is supported by the work of Smith et al. (1987), Magolski et al. (2013), Corbin et al. (2015), and Lucherk et al. (2016), who observed improvements in palatability traits with increases in marbling. For example, Corbin et al. (2015) found marbling to be the primary driver of beef flavor acceptability.

The genetic tests evaluated in the present study could be beneficial for use by producers who retain ownership at the feedlot because they might be able to use them to predict which animals will generate more revenue on a grid-based system by depositing more marbling. Research to compare carcass traits of purebred cattle to commercially available genetic panel scores to determine correlations, as well as validity of the genetic tests, has been completed in cattle of known genetic background (Quaas et al., 2007; DeVuyst et al., 2011). Additionally, the heritability of MS has been consistently reported as moderate when evaluated in beef [h^2^ = 0.67 (Mateescu et al., 2015); h^2^ = 0.43 (Minick et al., 2004); h^2^ = 0.37 (Utrera and Van Vleck, 2004)], and heritability of intramuscular fat content has been estimated to be high [0.93 (Shackelford et al., 1994)].

The sample population in the present study contained 73.9% Choice carcasses (Table 6). When dividing carcasses into High and Low groups based on their panel score, the High IT group contained 85.7% Choice carcasses and the High PG group contained 85.7% Choice. Additionally, the Low IT group contained 68.8% Choice, and the Low PG group contained 55.6% Choice. Using genetic tests, the current research was able to predict Choice cattle 85.7% of the time, thus increasing the percentage Choice in the present study by 11.8%. If producers were able to improve the percentage of cattle in their herd that produce carcasses of USDA Choice or better, they would be able to avoid discounts for failing to produce at least Choice beef (Smith, 2020).

**Table 6.** Frequency of quality grade within each marbling group

	Low IT	Low PG	High IT	High PG
Choice	11	5	6	12
Select	5	4	1	2
Total	16	9	7	14
% Choice	68.8	55.6	85.7	85.7

While there was an 11.8% increase in the percentage Choice observed with both tests, 12 of the 17 Choice carcasses were in the High PG group, but only 6 of the 17 Choice carcasses were in the High IT group. Therefore, a number of cattle that graded Choice were incorrectly placed in the Low group for both genetic panels. This would end up being costly for a producer deciding whether or not to retain ownership on a pen of cattle. More research is needed to determine if other groups of crossbred animals (i.e., different breed compilations, larger groups, and unrelated animals) have similar improvements in the percentage grading Choice, as well as a reduction in the number of animals in the low groups that end up grading choice. Since factors other than genetics, including nutrition, age, and animal handling influence MS, using these genetic tools does not guarantee that an animal will or will not grade Choice or better.

Commercially available genetic panel information for beef carcass quality traits have been shown to have a low, yet significant, correlation with objective carcass quality measurements in purebred animals (DeVuyst et al., 2011; Van Eenennaam et al., 2011a; 2011b). Van Eenennaam et al. (2011a; 2011b) predicted that genotyping would decline in price rapidly as more genomic information is gathered, and this has been realized over the last decade. They also predicted that the cost reduction will most likely result in an industry-wide adoption of the practice of using molecular breeding values, or values derived from genetic information to be used as a selection tool, to make breeding selections. When the analysis was conducted, the price of the Igenity test was $38/hd, which has since reduced in price to $29/hd (Neogen, Lincoln, NE). The decrease in price allows for more producers to adopt this technology, thus improving a producer’s opportunity to receive a premium for marbling, which would not only benefit the producer financially but would also benefit the consumer by providing a more consistent product and a better eating experience overall. Eventually, these commercially available genetic panel tests may become affordable to the point where commercial producers and feedlot operators use the tests on crossbred market cattle. This would allow managers to make feeding and marketing decisions and tailor implant strategies based on the individual animal’s potential to grade USDA Choice or better.

The Choice–Select spread is expected to continue to hit peaks over $20 seasonally for the foreseeable future (Zimmerman, 2020). When the Choice/Select spread is $5, a 900-lb carcass, which grades Select is $45 less valuable than if it grades Choice. In a group of 1,000 cattle weighing 900 lbs, which grade 73.9% Choice (pen average of the present study) versus 85.7% Choice (High IT/PG groups from the present study) at that price is $5,310. When the Choice/Select spread is $20, however, the improvement in that group of 1,000 cattle jumps up to $21,240. The cost of the genetic panels are currently $19 and $29, PredicGEN and Igenity, respectively. Therefore, current cost to test 1,000 head of cattle would be $19,000 and $29,000, respectively, and hard for the producer to justify even with a $20 spread. As these commercially available tests become more affordable, they could be used to separate cattle into groups based on their genetic potential and then managed accordingly.

## CONCLUSION

Based on these results, commercially available genetic tests could be a valuable tool for producers to be able to predict marbling by retaining ownership of feedlot steers with high genetic panel indexes. At times when the Choice–Select spread is high ($20), genetic panels could be cost effective for commercial producers to use at the feedlot level to make decisions about retaining ownership or for feedlot managers to make feeding, implant, and marketing decisions. Additionally, commercially available genetic panels cannot replace nutrition and proper animal handling practices. More research needs to be done to conduct a more robust economic analysis on this data to determine how producers can benefit financially from using these tests to make management decisions.
